# Reputational concerns, not altruism, motivate restraint when gambling with other people's money

**DOI:** 10.3389/fpsyg.2015.00848

**Published:** 2015-06-23

**Authors:** Kodi B. Arfer, Michael T. Bixter, Christian C. Luhmann

**Affiliations:** Psychology Department, Stony Brook UniversityStony Brook, NY, USA

**Keywords:** decision-making, altruism, evolutionary psychology, moral hazard, Bayesian inference

## Abstract

People may behave prosocially not only because they value the welfare of others, but also to protect their own reputation. We examined the separate roles of altruism and reputational concerns in moral-hazard gambling tasks, which allowed subjects to gamble with a partner's money. In Study 1, subjects who were told that their partner would see their choices were more prosocial. In Study 2, subjects were more prosocial to a single partner when their choices were transparent than when their choices were attributed to a third party. We conclude that reputational concerns are a key restraint on selfish exploitation under moral hazard.

## 1. Introduction

Nature is rich with social dilemmas, in which individuals must trade off between maximizing benefits to themselves and to others (Rand and Nowak, 2013). For example, the more a person donates to charity, the more others benefit and the less the donor has for herself. In fact, prosocial behavior of many kinds is surprisingly common (for reviews, see Sally, 1995; Fehr and Fischbacher, 2003). Why might people behave prosocially? One possible cause is a motivation to benefit others even at a cost to oneself, which we will call *altruism* (more precisely, “psychological altruism”; Wilson, 1992). Such altruism can be motivated by mental phenomena such as empathy (Batson, 1991), collectivism (Dawes et al., 1990), and inclusion of others in the self-concept (Cialdini et al., 1997). Counterintuitively, direct motivation to help others at one's own expense in this fashion may be evolutionarily adaptive (Fehr and Fischbacher, 2003). On the other hand, people may behave prosocially without altruism, that is, without such direct motivation. Populations can evolve a variety of means to force cooperation from selfish agents (Rand and Nowak, 2013). In human society, one such means is the law: the threat of imprisonment makes certain selfish behavior, such as theft, costly. A person with purely selfish motivation may still abstain from theft, appearing altruistic, purely to avoid this punishment. Thus, given any prosocial choice a person has made, it is ambiguous how much the choice was motivated by altruism and how much by shrewd self-interest.

### 1.1. Reputational concerns

It has been argued that improving one's reputation is a particularly important way that prosocial behavior can ultimately serve self-interest (Fehr and Fischbacher, 2003). Experimental work is consistent with this view. For example, Sylwester and Roberts (2010) had subjects play two rounds of an economic game. Subjects who were more generous in the first round were chosen by partners who were more generous in the second round. By this means, subjects who were more generous initially earned more overall. Sylwester and Roberts reasoned that more generous subjects had invested in their reputation.

Bereczkei et al. (2007) attempted to distinguish the direct effect of reputational concerns on generosity from altruistic motives. Students were asked in the presence of their classmates to volunteer for charities. When commitments to volunteer were made publicly, so that classmates could observe them, the proportion of subjects who volunteered jumped from 25 to 50%. Thus, Bereczkei et al. inferred that the observed difference in generosity was due to reputational concerns. Similar findings of audience-dependent generosity have been obtained by other investigators, particularly among subjects who are especially self-interested (“egoistic”; Simpson and Willer, 2008) or socially manipulative (“Machiavellian”; Bereczkei et al., 2010).

There is also evidence that people will take opportunities to appear prosocial without actually being so. In the ultimatum games of Kagel et al. (1996), subjects who knew their own payoff from a deal would be tripled but who knew their opponent did *not* know this typically offered an even split. Thus, subjects behaved in a way that appeared fair (perhaps to increase the odds the opponent would accept the split) but was actually self-interested. In the dictator games of Dana et al. (2007), subjects were more likely to choose a selfish split when the opponent did not know the payoffs. Hence, subjects acted less prosocially when their opponents could not evaluate how prosocial they were being. However, these findings are difficult to interpret with respect to reputation management, because in both studies, it is not clear that subjects could identify each other, and thus, reputational consequences were not clearly involved.

### 1.2. Moral hazard

In the bulk of the experimental tasks described above, there was no uncertainty other than subjects' ignorance of other people's thoughts and plans. Real-life social dilemmas, on the other hand, are often uncertain. Taking advantage of other parties may or may not ultimately harm them. One class of social dilemma which captures this kind of risk is moral hazard (more precisely, “indirect moral hazard,” Crosby, 1905, or “morale hazard,” McLeman and Smit, 2006). A person is said to be under moral hazard when potential negative consequences of her decisions will be borne by a third party, regardless of whether the situation would be seen as morally charged according to psychological or philosophical notions of morality. Moral hazard has primarily been studied in the context of insurance (e.g., Grossman, 1992; Quiggin et al., 1993; Abraham et al., 2010). The provision of insurance shifts losses from an individual to her insurance agency, increasing her incentives for risk-taking and therefore possibly making her less sensitive to risk. For example, people who are insured against floods are under moral hazard because they face less potential loss by purchasing property in a flood-prone area (e.g., Burby, 2001; Huber, 2004; Bagstad et al., 2007). However, moral hazard also arises in everyday situations. A person may be less careful to lock her friend's door than her own, because she does not experience the negative consequences of a robbery. Similarly, an HIV-positive person having sex with an HIV-negative partner has less to lose by neglecting to use a condom. More broadly, moral hazard is related to other means of exploiting the contributions of others, such as social loafing (Karau and Williams, 1993), as well as to differences in risk-taking between individuals and groups (Zajonc et al., 1969; Burnstein et al., 1973; Gardner and Steinberg, 2005).

Recently, we (Bixter and Luhmann, 2014) experimentally demonstrated increased risk-taking under moral hazard. Subjects were asked to choose between a mixed gamble (e.g., a 70% chance of gaining $40 and a 30% chance of losing $40) and a sure gain (e.g., $15). Each trial had a different gamble; gambles came in various types. For standard gambles, subjects had to bear the full loss themselves if they chose the mixed gamble and lost. For shared gambles, half of any loss (e.g., $20 of the $40) would be borne by a third party, the subject's partner. Thus, shared gambles were more beneficial to the subject, but more harmful to the partner, than standard gambles. As in any moral-hazard situation, taking a shared gamble, compared to an equivalent standard gamble, meant reducing one's own potential loss by an amount equal to the potential loss inflicted upon someone else. This situation is similar to a dictator game, where one can increase one's own gain by reducing another person's gain by the same amount (Forsythe et al., 1994). Altruistic subjects, by refraining from shared gambles, could thus benefit their partner (by protecting her from loss) while forgoing extra gain to themselves. Other trials in Bixter and Luhmann (2014) involved matched gambles, which involved the same loss to the subject as the corresponding shared gamble (e.g., a 70% chance of gaining $40 and a 30% chance of losing $20) but no loss to the partner. A subject entirely insensitive to her partner's welfare should then treat matched gambles identically to shared gambles. The results of the study indicated that subjects were more likely to take shared gambles and matched gambles than standard gambles; in fact, no significant difference was found between the former two. That is, subjects' behavior was consistent with a total disregard for the welfare of others.

In the second experiment presented in Bixter and Luhmann (2014), we tried to increase subjects' prosocial behavior by decreasing the social distance between subject and partner—that is, by making subjects feel personally closer to their partner. Previous research (e.g., Jones and Rachlin, 2006) had found that people are more generous toward others to whom they feel closer. Though there was a suggestion that shared gambles were less attractive than matched gambles, this difference was not significant. Overall, the results were similar to that of the first experiment.

The present studies are another attempt to induce prosocial behavior in this kind of moral-hazard gambling task. We investigated if reputational concerns can reduce subjects' willingness to take shared gambles.

## 2. Study 1

Study 1 was a two-condition between-subjects experiment. In the Visible condition, we made reputational concerns salient by telling subjects that their partner would see what choices they had made, and how they had affected the partner's welfare. In the Anonymous condition, we told subjects that their choices would be kept secret from the partner. We expected subjects in the Visible condition to be less willing to take advantage of the partner under moral hazard.

### 2.1. Method

For both Study 1 and Study 2, our research protocol was approved by the Committees on Research Involving Human Subjects of Stony Brook University's Office of Research Compliance, and all subjects provided informed consent.

#### 2.1.1. Subjects

Subjects were 38 undergraduates at Stony Brook University run in pairs. There were 19 subjects in each of the Anonymous and Visible experimental conditions (which were assigned at random per subject without regard to dyads). All subjects were native speakers of English. There were 5 female–female dyads, 14 opposite-gender dyads, and no male–male dyads. See Table 1 for additional demographic characteristics. Subjects received partial course credit and $5 for participation.

**Table 1:** **Frequency tables of demographic characteristics for the subjects in Study 1, grouped by experimental condition**.

	**Anonymous**	**Visible**
Sample size	17	17
Gender		
Female	10	11
Male	7	6
Age		
18	10	6
19	3	0
20	3	4
21	0	2
22	1	3
23	0	1
27	0	1
Year in college		
1st	11	6
2nd	2	0
3rd	4	5
4th	0	4
5th or above	0	1
No response	0	1
Race and ethnicity		
Asian	4	4
Black	1	1
Native American	1	0
White	9	8
White and Hispanic	1	2
Multiracial	0	1
Multiracial and Hispanic	0	1
Other	1	0
Is bilingual		
No	13	13
Yes	4	4
Sexual orientation		
Heterosexual	14	15
Homosexual	1	0
Bisexual	1	1
No response	1	1
Dominant hand		
Left	2	1
Right	15	16
Has normal visual acuity		
No	2	0
Yes	15	17

#### 2.1.2. Gambling task

The gambling task was an adaption of the moral-hazard gambling task discussed earlier (Bixter and Luhmann, 2014). Subjects gambled with hypothetical money, but were instructed that they would receive $5 in real money if their total earnings reached a certain unspecified threshold. Such an incentive scheme, following Bixter and Luhmann (2014), implied that every trial was in some sense realized (subjects could not be assured that only one of their decisions would end up affecting their and their partner's welfare) but also that subjects' choices could not be affected by how much they had earned mid-task, because they did not know the threshold. In fact, we paid the $5 to every subject, so the threshold was an arbitrarily low number.

On each trial, the subject chose between a sure gain of $15, presented at the top of the computer screen, and a mixed gamble, presented at the bottom, by pressing an arrow key. The subject was instructed that for some, specially indicated gambles, any losses would be shared with her partner, another subject she had seen earlier. Subjects were informed that they were not vulnerable to sustaining losses from their partner's decisions.

**Figure 1:**
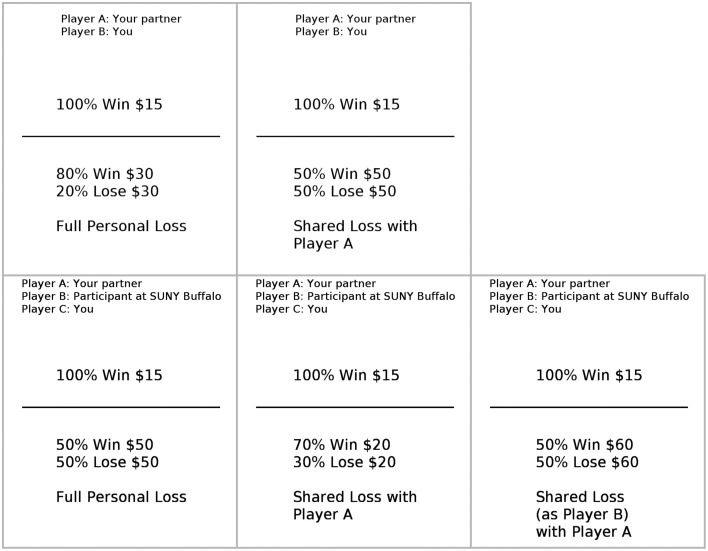
**Sample trials, as they were displayed to subjects**. A reminder of player assignments always appeared at the top of the screen. **Upper row**: Trials from Study 1. On the left is a standard gamble, and on the right is a shared gamble. **Lower row**: Trials from Study 2. On the left is a standard gamble; matched gambles appeared the same, except with the loss amount halved. In the center is a local shared gamble; remote shared gambles appeared the same, except reading “Shared Loss with Player B.” On the right is a deceit gamble.

All gambles had two outcomes, winning and losing. There were three types of gambles (see Figure 1, upper row; catch gambles appear similarly to standard gambles and hence are not shown).

- For standard gambles, the probability of winning was 50, 60, 70, or 80%, and the amount to be won or lost was $20, $30, $40, $50, or $60.
- Shared gambles were like standard gambles except if the subject took the gamble and lost, half the loss amount was ostensibly inflicted on the partner instead of the subject. Thus, shared gambles entailed moral hazard.
- Catch gambles were like standard gambles, but the amount to be won or lost was $10. Thus, the sure gain of $15 strictly dominated all catch gambles. Selection of a catch gamble was taken as evidence of thoughtless responding.

Subjects were offered every combination of probability, amount, and gamble type twice (except that catch gambles were presented only once each). This arrangement yielded a total of 84 trials, which were presented in a random order. When a gamble was selected, the outcome (whether the subject won or lost) was not shown during the task, because we did not want outcomes to influence subsequent choices.

#### 2.1.3. Procedure

The experimenter met both subjects of each dyad together and explained the gambling task orally and with a handout. (See Appendix A in Supplementary Material for the full text of the handout.) Subjects were told the task had two roles, Player A and Player B. Player B would be offered shared gambles (which they could take to benefit themselves at the chance of causing loss to Player A), whereas Player A would not be offered any shared gambles. Thus, Player B could causes losses to Player A, but not vice versa. Subjects were told they would be randomly assigned to roles. In reality, all subjects were assigned to the role of Player B. We used this deception (and a similar one in Study 2) because our interest was in the behavior of the most powerful player, so having real subjects take on less powerful roles would have wasted subjects.

After this meeting with the experimenter, the two subjects of each dyad went to adjacent private rooms to complete the task on computers. The computer told each subject that they were Player B and then displayed a message depending on a randomly assigned experimental condition. In the Anonymous condition, the message was “Your partner will not be told what choices you made or whether you affected their chances of winning $5.” In the Visible condition, the message was “After the task is complete, your partner will see a complete list of the choices you made and how you affected their chances of winning $5. The two of you will then have the opportunity to discuss your choices.” This message was the only manipulation that depended on condition.

After subjects completed the gambling trials, their total earnings from the gambling task were displayed, and they were paid $5 of real money. (This $5 was the same $5 mentioned above as compensation.) The behavior of subjects in the Visible condition was not actually shown to other subjects. We think it would have been unethical to do so, since showing a subject the shared gambles taken by her partner could be upsetting to both subjects, and we had no plan to collect useful data from such an exchange.

### 2.2. Results and discussion

See http://arfer.net/projects/hazard for raw data, task code, and analysis code for both Study 1 and Study 2.

Four subjects accepted 2 or more catch gambles. These subjects were excluded from further analysis, as were all catch trials. The remaining sample was also evenly split by condition, with 17 subjects in each.

**Figure 2:**
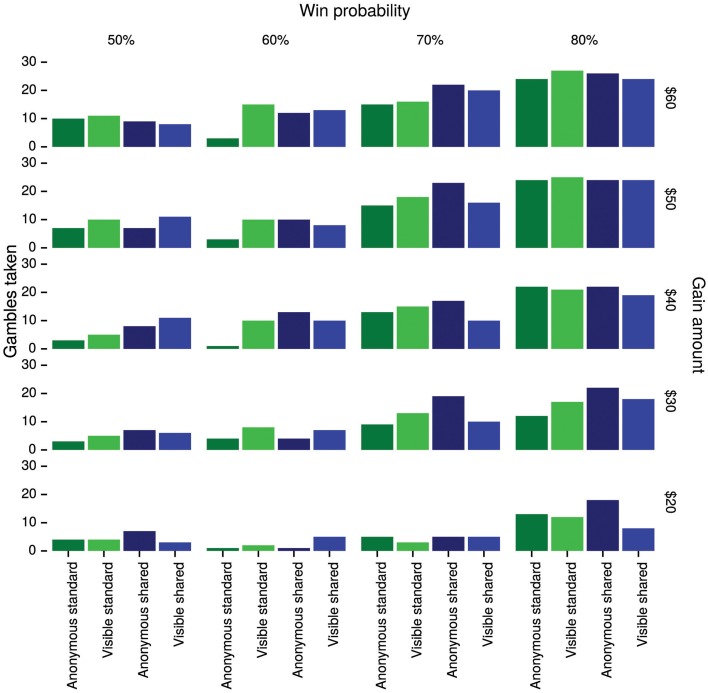
**The number of gambles taken in Study 1 by condition, gamble type, gain amount, and win probability**. Since each gamble was offered twice, each subject may contribute up to two units to each bar.

Overall, Anonymous subjects took 28% of standard gambles and 41% of shared gambles, whereas Visible subjects took 36% of standard gambles and 35% of shared gambles. See Figure 2 for a complete breakdown of choices collapsed across subjects.

We analyzed the effects of gamble type and experimental condition on gambling behavior with Bayesian mixed-effects logistic regression. We chose regression over ANOVA or *t*-tests to avoid treating numerical predictors, such as gamble probability, as categorical, and we chose logistic regression over linear regression because our dependent variable, whether the subject took the gamble, was dichotomous. We used Bayesian methods rather than null-hypothesis significance testing chiefly so that we could describe precisely what could be inferred about model parameters, properly accounting for all sources of uncertainty tracked by the model. Doing Bayesian data analysis is also consistent with growing dissatisfaction with standard uses of significance testing (e.g., Cohen, 1994; Gelman and Stern, 2006; Kruschke, 2010; Wagenmakers et al., 2011; Cumming, 2014).

For the dependent variable, we coded taking the gamble as 1 and taking the sure gain of $15 as 0. The model had a single per-subject random effect drawn from a normal distribution with mean 0 and standard deviation σ, where the hyperparameter σ was given a prior density uniform on [1/10, 100]. Fixed effects are listed in Table 2. All fixed effects were assigned improper uniform priors. Notice that the model does not include parameters for loss (or expected value). This means that the values of b_g_shared_anon and b_g_shared_visible will include the effect of reduced loss to the subject as well as increased loss to the partner, so our analyses will not answer the question of whether subjects would make prosocial choices at no cost to themselves. (The effect of reduced loss alone is examined with matched gambles in Study 2.)

**Table 2:** **Fixed effects of the regression model in Study 1**.

**Parameter**	**Predictor**
**PARAMETERS OF INTEREST**	
b_g_shared_anon	Whether this gamble is shared and the subject is Anonymous
b_g_shared_visible	Whether this gamble is shared and the subject is Visible
**NUISANCE PARAMETERS**	
b0	None (constant term)
b_g_egain	Product of win probability and gain amount
b_female	Whether the subject is female
b_local_female	Whether the local partner is female

Model parameters were estimated with the Markov chain Monte Carlo sampler Stan (http://mc-stan.org). Markov chain Monte Carlo is a numerical method for estimating the parameters of a Bayesian model. In accordance with the recommendations of Gelman et al. (2004), eight chains were run with random initial values, and for each chain, 250 adaptive burn-in iterations were discarded and 250 non-adaptive sampling iterations were kept, for a total of 2000 samples per parameter. This resulted in a Gelman-Rubin diagnostic (Gelman and Rubin, 1992) of less than 1.05 for all parameters.

**Figure 3:**
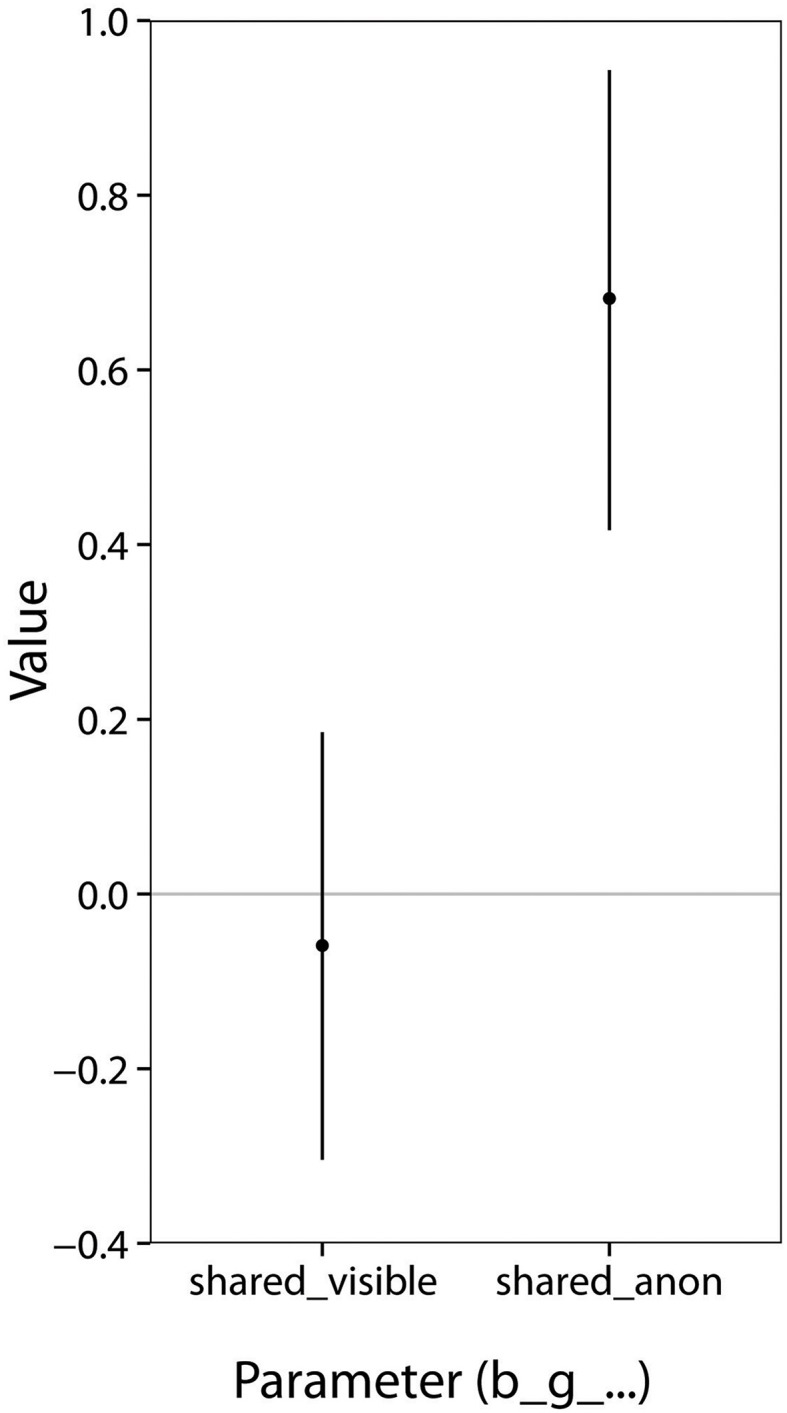
**Posterior means and 95% equal-tailed credible intervals for the effect of each gamble type in Study 1**. The *y*-axis is in logit units, so a value of 1 implies a 2.7-fold increase in the odds of taking a gamble.

Parameter estimates are shown in Figure 3. With posterior samples in hand, to answer the question of how the experimental condition affected subjects' willingness to take shared gambles, we computed the posterior probability that b_g_shared_anon is greater than b_g_shared_visible. This inequality was satisfied by every sample, so the posterior probability that it is true exceeds 99%. We conclude that subjects were indeed less willing to take shared gambles, exposing their partner to risk, when their reputation was at stake. In fact, in the Visible condition, it is more likely than not that subjects preferred standard gambles to shared gambles (specifically, the posterior probability of b_g_shared_visible being less than 0 is 68%), despite how a standard gamble has twice the potential loss to the subject as a shared gamble, suggesting subjects preferred increased monetary losses to themselves over reputational fallout.

## 3. Study 2

In Study 1, we found that reputational concerns can induce restraint of risk-taking under moral hazard. Specifically, telling subjects that their partner would be able to confront them about how they had gambled with the partner's money reduced such gambling. Study 2 used a more complex task, with two partners, to examine how reputation effects interact with social-distance effects.

In Study 2, there was no between-subjects manipulation, but we offered subjects shared gambles with two partners. The *local partner*, as in Study 1, was another subject. Since Bixter and Luhmann (2014) seemed to have failed to decrease social distance enough to make subjects choose more prosocially, we used a stronger manipulation of social distance: we introduced subjects to each other and attempted to establish rapport. The *remote partner*, by contrast, was an unseen and unnamed person. As in the Visible condition of study 1, subjects were told their partners would be able to see what losses they had incurred from the subject's actions.

We included three kinds of shared gambles: local shared gambles (which shared losses with the local partner), remote shared gambles (which shared losses with the remote partner), and deceit gambles (which shared losses with the local partner while protecting the subject's reputation). If subjects were genuinely altruistic toward the local partner (i.e., cared about the local partner's welfare), they should have found deceit gambles no more attractive than local shared gambles, since both expose the same partner to loss. Subjects behaving prosocially out of reputational concerns alone, on the other hand, should have found deceit gambles more attractive than local shared gambles, because deceit gambles allow subjects to benefit from shared gambles without suffering reputational fallout.

Finally, Study 2 also included matched gambles, as in Bixter and Luhmann (2014). Matched gambles were like standard gambles but with the loss amount halved, so that the potential loss to the subject from taking a matched gamble was the same as from taking a shared gamble. (The potential gain, and the probabilities of gain and loss, were the same between matched and shared gambles as well, but these were equal among all gamble types. Together, these facts imply that a matched gamble had not just the same expected value for the subject as a shared gamble, but the identical probability distribution of outcomes for the subject.) Thus, subjects who were entirely indifferent to the welfare of their partners should have treated matched gambles the same as shared gambles.

### 3.1. Method

#### 3.1.1. Subjects

Subjects were 34 undergraduates at Stony Brook University run in pairs. All subjects were native speakers of English. There were 4 male–male dyads, 4 female–female dyads, and 9 opposite-gender dyads. Ages ranged from 18 to 26. Subjects received partial course credit and $5 for participation.

#### 3.1.2. Tasks

##### 3.1.2.1. Relationship closeness induction task

Dyads completed a form of the Relationship Closeness Induction Task (RCIT; Sedikides et al., 1999). The RCIT is similar to the method of inducing interpersonal closeness described by Aron et al. (1997), but much shorter. Subjects were given lists of questions to ask each other and answer. Questions become progressively more intimate over the course of the task, ranging from “Where are you from?” to “What would be the perfect lifestyle for you?” to “What's your most frightening early memory?.” Subjects were allowed to finish all the questions at their own pace. The task took 10–15 min per dyad.

##### 3.1.2.2. Gambling task

In Study 2, unlike Study 1, subjects were told there were two partners: the “local partner,” the subject's dyadic counterpart, and the “remote partner,” an unseen and unnamed other person at another university. In reality, the remote partner did not exist.

Whereas Study 1 offered one type of shared gamble, Study 2 offered three (see Figure 1, lower row). All ostensibly inflicted half the loss amount on a third party. Which third party was exposed to loss depended on the gamble type.

- Local shared gambles exposed the local partner to loss.
- Remote shared gambles exposed the remote partner to loss.
- Deceit gambles exposed the local partner to loss (like local shared gambles); however, any loss sustained by the local partner would be attributed to the actions of the remote partner rather than the subject.

Study 2 also had an additional gamble type, the matched gamble. Matched gambles were like standard gambles but with the loss amount halved. Thus, subjects who were entirely indifferent to the welfare of their partners should have treated matched gambles the same as shared gambles.

In summary, Study 2 had six types of gambles: standard, catch, matched, local shared, remote shared, and deceit shared. This assortment differed from what was offered in Study 1 in the addition of matched gambles and in the replacement of the one kind of shared gamble with three kinds of shared gambles.

Subjects were offered every combination of probability, amount, and gamble type (only once), for a total 104 trials.

##### 3.1.2.3. Distance-ranking task

Since we expected that interpersonal closeness would influence prosocial behavior, we had subjects rank 12 people, including their two partners, in order of social distance. First, subjects were asked to think of a particular instance of, and provide a nickname for, each of several types of people: “Your best friend,” “The parent or stepparent you're closest to,” “A friend of a friend,” “A childhood friend you haven't spoken to in years,” “A relative you see no more than a few times a year,” “Your primary physician,” “A cashier at a store (or a university eatery) that you go to often,” “A classmate you can recognize but you've never spoken to,” “A relative who you know a least a little about but who died before you were born,” and “A stranger you've seen once or a few times and who you know nothing about,” as well as the local and remote partners. Then subjects were shown a list of just their provided nicknames and asked to “sort these people according to how close you feel to them.” We coded the closest person as 1 and the furthest as 12.

#### 3.1.3. Procedure

First, subjects completed the RCIT, which was described as a new communication task. Then the experimenter explained the gambling task orally and with a handout. (See Appendix B in Supplementary Material for the full text of the handout.) Subjects were told the task had three roles, Player A, Player B, and Player C, who differed in the shared gambles they would be offered. Player A would see no shared gambles, Player B could share losses with Player A but not Player C, and Player C could share losses with Players A and B. Subjects were told they would be randomly assigned to roles. In reality, all subjects were assigned to the role of Player C, and were told upon seeing their own assignment that that the remote partner (labeled “Participant at SUNY Buffalo”; see Figure 1) would be Player B and the local partner (labeled “Your partner”) would be Player A.

Only when subjects began the task in adjacent private rooms was the existence of deceit gambles revealed, because we did not want subjects to think that their local partner knew deceit gambles existed and thus would not be entirely deceived. After subjects completed the gambling task, they performed the distance-ranking task. Finally, their total earnings from the gambling task were displayed, and they were paid $5 of real money.

### 3.2. Results and discussion

Two subjects accepted 2 or more catch gambles. These subjects were excluded from further analysis, as were all catch trials.

**Figure 4:**
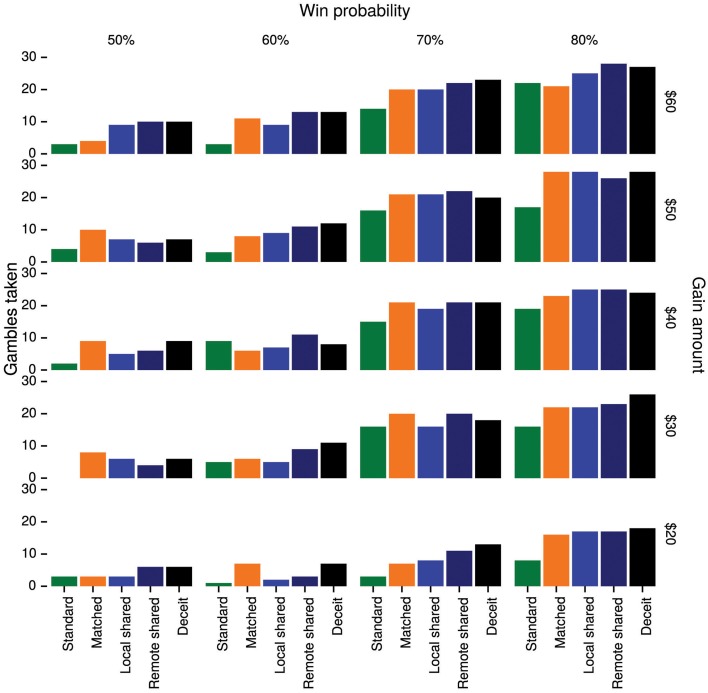
**The number of gambles taken in Study 2 by gamble type, gain amount, and win probability**.

Overall, subjects took 28% of standard gambles, but 42% of matched gambles, 41% of local shared gambles, 46% of remote shared gambles, and 48% of deceit gambles. See Figure 4 for a complete breakdown of choices collapsed across subjects.

The median difference between social-distance ranks of the local and remote partners was 3, with the local partner ranked closer. This difference, being on a 12-point scale, was smaller than might have been expected; however, the rated social distance of the remote partner appeared to suffer from a strong ceiling effect, with 84% of subjects selecting ranks 10, 11, or 12.

We analyzed the effects of gamble type and rated social distance on gambling behavior with a Bayesian mixed-effects logistic regression, as in Study 1. The fixed effects for the Study 2 model are shown in Table 3. Since social-distance rank of the remote partner appeared limited by a ceiling effect, this variable was not included as a predictor. We generated posterior samples as in Study 1.

**Table 3:** **Fixed effects of the regression model in Study 2**.

**Parameter**	**Predictor**
**PARAMETERS OF INTEREST**	
b_g_matched	Whether this gamble is a matched gamble
b_g_shared_local	Whether this gamble is a local shared gamble
b_g_shared_remote	Whether this gamble is a remote shared gamble
b_g_shared_deceit	Whether this gamble is a deceit gamble
**NUISANCE PARAMETERS**	
b0	None (constant term)
b_g_egain	Product of win probability and gain amount
b_female	Whether the subject is female
b_local_female	Whether the local partner is female
b_both_female	Whether both are female
b_local_distrank	Social distance rank of the local partner

#### 3.2.1. Effect of gamble types

First, we expected all four gamble-type parameters—b_g_matched, b_g_shared_local, b_g_shared_remote, and b_g_shared_deceit—to exceed zero, indicating that subjects were more likely to take matched or shared gambles than standard gambles. We also examined whether b_g_shared_deceit was greater than b_g_shared_local, which would indicate that subjects were more willing to cause loss to the local partner when their reputation was not at stake. Finally, we checked that b_g_shared_remote exceeded b_g_shared_local, in accordance with greater social distance leading to decreases in prosocial choices.

**Figure 5:**
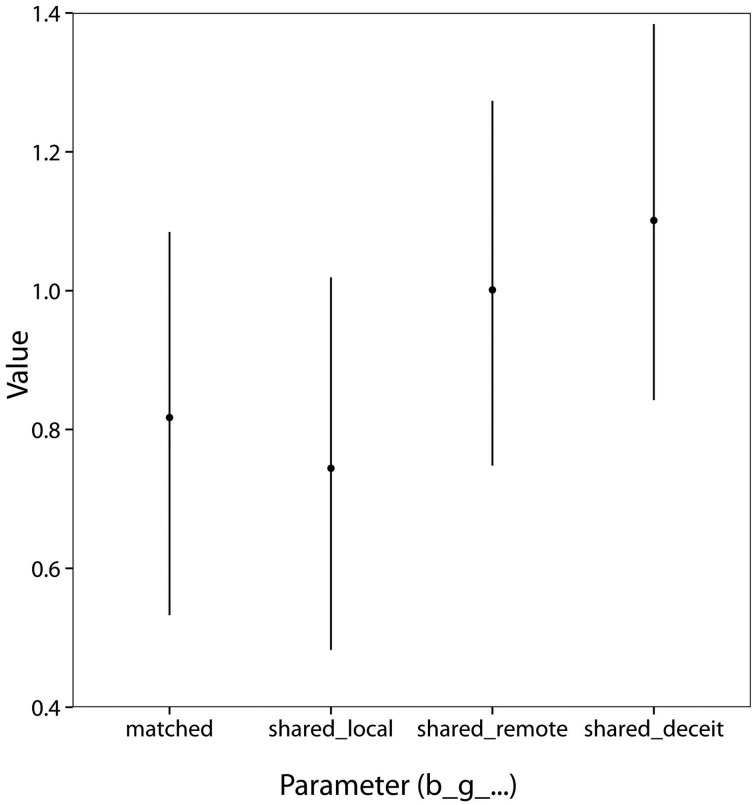
**Posterior means and 95% equal-tailed credible intervals for the effect of each gamble type in Study 2**. The *y*-axis is in logit units, so a value of 1 implies a 2.7-fold increase in the odds of taking a gamble.

Parameter estimates are shown in Figure 5. We assessed the effects of the various gamble types by comparing the values of the corresponding parameters. As can be seen in Table 4, the posterior probability of each parameter being greater than zero exceeded 99%, indicating that matched gambles and all kinds of shared gambles were more appealing to subjects than standard gambles. Critically, with probability exceeding 99%, deceit gambles were more appealing than local shared gambles, indicating subjects were more willing to expose local partners to loss when disguised as a third party and thus protected from reputational consequences. Furthermore, with probability 97%, remote shared gambles were more appealing than local shared gambles, indicating that generosity decreased with social distance.

**Table 4:** **Probabilities of inequalities between gamble-type parameters**.

	**b_g_shared_remote**	**b_g_shared_local**	**b_g_matched**	**zero**
b_g_shared_deceit	0.781	0.998	0.991	1.000
b_g_shared_remote		0.974	0.906	1.000
b_g_shared_local			0.298	1.000
b_g_matched				1.000

*Each cell gives the probability that the row parameter is greater than the column parameter*.

Two other more counterintuitive results should also be noted. First, deceit gambles (probability 99%) and remote shared gambles (probability 91%) were more appealing than matched gambles, although they did not afford subjects any additional protection from loss. Possibly these kinds of shared gambles had some appeal above and beyond their reduced potential loss. Second, there was a suggestion (probability 78%) that deceit gambles were preferred to remote gambles. Perhaps subjects' concern for their reputation with the remote partner plus their genuine interest in the remote partner's welfare was greater than their genuine interest in the local partner's welfare. Genuine interest for the local partner plus concern for reputation with the local partner, however, may have made local shared gambles less appealing than matched gambles (probability 70%).

## 4. General discussion

We examined altruism, reputational concerns, and social distance as causes of prosocial behavior. Subjects were offered a series of gambles, some of which allowed them to share any losses with a partner. In Study 1, we found that making reputational concerns salient made subjects take fewer gambles that would expose their partner to loss. In Study 2, subjects were less willing to openly expose a partner they had met to loss (by taking local shared gambles) than a partner they hadn't met (by taking remote shared gambles). But when subjects could pseudonymously expose the local partner to loss (by taking deceit gambles), this apparent concern disappeared. In fact, subjects took more deceit gambles than remote shared gambles. Therefore, the apparent concern for the local partner's welfare can be explained by reputational concerns alone. Together, Studies 1 and 2 suggest reputational concerns are a key motive for restraint of risk-taking under moral hazard.

### 4.1. Social distance

Our subjects' greater willingness in Study 2 to take remote shared gambles than local shared gambles is consistent with past work on social distance. As the social distance between a subject and a third party decreases, generosity increases (Jones and Rachlin, 2006). Manipulations to decrease social distance that have increased generosity in economic games include allowing subjects to see each others' faces (Bohnet and Frey, 1999) and revealing last names (Charness and Gneezy, 2008). By contrast, our earlier study using a similar moral-hazard paradigm (Bixter and Luhmann, 2014) found no significant effect of whether subjects had personally met their partner. Our strengthened design, which involved an opportunity to become socially closer to the local partner (the RCIT) and an especially distant remote partner for contrast, found an effect of social distance that Bixter and Luhmann (2014) did not. The importance of social distance for economic decision-making is further suggested by its effects on variables other than generosity. Ziegler and Tunney (2012), for example, found that subjects made more patient choices on the behalf of third parties the less genetically related the third party was to the subject.

### 4.2. Additional findings

We found that most kinds of shared gambles were preferred to standard gambles. This is the basic moral-hazard effect of people weighting losses to others less than losses to themselves, replicating Bixter and Luhmann (2014). On the other hand, Visible subjects in Study 1, for whom reputational concerns were particularly salient, may have preferred standard gambles to shared gambles, suggesting that a personal loss may be preferred to facing blame for causing a loss to somebody else.

Our most surprising finding was subjects' preference in Study 2 for deceit gambles over matched gambles (and their less likely preference for remote shared gambles over matched gambles). Effectively, these shared losses were treated as gains. Possible explanations include misery loving company (e.g., Cooper and Rege, 2011); a dissonance effect creating antipathy toward partners (Glass, 1964); an antisocial desire to punish the remote partner (Branas-Garza et al., 2014) by harming her reputation, on the presumption that the remote partner has taken shared gambles harming the local partner; or a view of the deceit gambles as special opportunities that should be taken advantage of.

### 4.3. Moral hazard

There are various ways a person can behave prosocially toward another party. A person can perform an action that incurs a personal cost while simultaneously increasing the welfare of the third party. The real-world action that best represents this form of prosociality is charitable donation. However, people can also behave prosocially toward another party by refraining from committing an action that benefits themselves but decreases the welfare of the third party. Moral hazard is a situation that captures this latter scenario, because increased risk-taking under moral hazard can lead to personal benefits while exposing another party to losses that she is not directly responsible for.

Moral hazards are pervasive throughout the real world and can lead to large societal costs (e.g., Blanchard-Boehm et al., 2001; Okamoto, 2009). As a result, it is important to gain a better understanding of the processes that affect people's decision making when they are under moral hazard. Furthermore, research in the laboratory has overwhelmingly focused on the former class of prosocial behavior, such as allotments in ultimatum and dictator games (Camerer and Thaler, 1995; Hoffman et al., 1996; Charness and Gneezy, 2008). The experiments of Leliveld et al. (2009), however, suggest that people care more about fairness and less about self-interest when losses rather than gains are at stake, and Zhou and Wu (2011) found a higher rate of altruistic punishment in ultimatum games with losses than in ultimatum games with gains. Clearly, in order to achieve a better understanding of the origins of prosociality, it is necessary to study instances of prosocial behavior across a variety of contexts and situations. The results of our studies suggest that reputational concern is one available method to increase prosocial behavior when a decision-maker is under moral hazard.

### 4.4. Limitations

As just discussed, there are many kinds of situations to which questions of altruism and prosocial behavior apply. Our studies focused on moral hazard, which is only one such kind of situation, and therefore they do not say much about altruism in other situations. Furthermore, it is believable that people behave differently in different contexts of moral hazard. For example, perhaps people are less prosocial toward insurance agencies than individuals, or perhaps people are more prosocial when exploitation would endanger human health rather than finances. Future research would need to examine such moderating effects directly in order to discover their influence.

In our studies, subjects were placed in an implicit position of power by being offered opportunities to exploit partners. Such an opportunity is part of the definition of moral hazard, but our task differed from most real-life moral-hazard situations in that exploitation was presented as an explicit option. Thus, by a sort of demand characteristic driven by the perceived legitimacy of exploitation, subjects may have been more willing to take shared gambles than they would been in an equivalent real-life situation. Fortunately, any such demand characteristic would apply equally to the two conditions of Study 1 and to all the kinds of shared gambles in Study 2, meaning that the contrasts of chief interest cannot have been affected.

Our studies used an unusual incentive scheme that paid subjects real money if they had reached an unspecified threshold of in-task earnings, as opposed to, for example, paying subjects in direct proportion to in-task earnings, or choosing a random trial to realize. This scheme has the advantage of making every trial count without allowing subjects to exploit dependencies between gambles or “play with the house's money.” On the other hand, it complicates comparison to other studies. Future research may benefit from standardizing on compensation.

In Study 2, although we attempted to create social closeness between subjects with the RCIT, and the distance-ranking task confirmed that subjects generally saw the remote partner as more distant than the local partner, subjects still did not feel very close to the local partner. After all, a short in-lab exercise between strangers cannot be expected to forge a social bond comparable in magnitude to long-term friendships or romantic relationships. As mentioned in the introduction, research on social discounting (e.g., Jones and Rachlin, 2006) shows that people are willing to make larger sacrifices to benefit a third party the closer they are to the third party. Thus, our results leave open the possibility that subjects will be more altruistic under moral hazard when the third party whose resources they can gamble with is a true intimate.

### 4.5. Self-interest vs. prosocial behavior

In previous studies, subjects could directly, materially gain from maintaining a reputation (Kagel et al., 1996; Simpson and Willer, 2008; Sylwester and Roberts, 2010) or at least expected to repeatedly interact with the audience in the future (Bereczkei et al., 2007, 2010). In our studies, by contrast, the advantage to maintaining a good reputation in the eyes of partners was less clear, since subjects had no reason to believe they would interact with their partners again. We suggest, then, that people inherently value reputation.

We found that subjects were self-interested but not transparently so. This observation is an example of how prosocial behavior is not, in general, incompatible with the notion that organisms are largely self-interested. On the contrary, self-interest can be useful as an explanation for prosocial behavior.

### Conflict of interest statement

The authors declare that the research was conducted in the absence of any commercial or financial relationships that could be construed as a potential conflict of interest.
