# Supplementary material for: Reputational concerns, not altruism, motivate restraint when gambling with other people's money
Source: Front Psychol. 2015 Jun 23;6:848. doi: 10.3389/fpsyg.2015.00848 (PMC4477537; doi:10.3389/fpsyg.2015.00848)
Supplement: Supplementary file 1 [file AppendixA.PDF]

Appendix B

Instructions

In this study, you will be offered a series of gambles. At the end of the experiment, if you've accumulated a certain unspecified amount of money in the task, you will be paid $5 in real money.

For each trial, you'll choose between a sure gain of $15 and a gamble. For example, a trial might look like this:

```
100% Win $15
____________

70% Win $50
30% Lose $50
```

This represents a choice between $15 and a gamble in which you are 70% likely to win $50 and 30% likely to lose $50.

The computer will keep track of your winnings across all trials. So for the above trial, if you chose the top option, $15 would be added to your total. If you chose the bottom option and you won, $50 would be added. However, if you chose the bottom option and lost, $50 would be subtracted from your total. Nothing special will happen if your total goes below $0 (the task will continue normally).

Now, there are three players in this task: Player A, Player B, and Player C. There are three altogether because there are two players in this lab and one other player in the Duane Lab in SUNY Buffalo's psychology department. The computer will choose randomly who will be Player A, Player B, and Player C.

If you are **Player A**, you will only see trials like the one described above.

If you are **Player B**, you will also see a second type of trial. Your choices on these trials can have consequences for both you and Player A. One of these trials might look like this:

```
100% Win $15
________________

70% Win $50
30% Lose $50

Shared Loss with
Player A
```

This trial is similar to the one described above, but the loss associated with the gamble will be shared evenly between both Player A and Player B. So if Player B took the gamble and won, $50 would be added to Player B's own winnings. On the other hand, if Player B took the gamble and

lost, $50 would be lost, but only half of it (that is to say, $25) would be subtracted from Player B's total. The other $25 would be subtracted from Player A's total. This kind of gamble will be labeled "Shared Loss with Player A". The first kind of gamble, in which all losses will be subtracted from Player B's winnings, will be labeled "Full Personal Loss".

If you are **Player C**, then, like Player B, you will see both "Full Personal Loss" trials and "Shared Loss with Player A" trials. The difference is that you will also see "Shared Loss with Player B" trials.

So, in summary:

- Player A can never choose to share losses, but may lose money from Player C's or Player B's shared-loss trials.
- Player B can choose to share losses with Player A, but may lose money from Player C's shared-loss trials.
- Player C can choose to share losses with Player A and can choose to share losses with Player B, but cannot lose money because of other players' choices.

On every trial, choices are made by pressing the **up** and **down** arrow keys. If you prefer the sure gain of $15 (which is always the top option), press the **up** arrow key. If you prefer the gamble (which is always the bottom option), press the **down** arrow key.

Remember that whether each player receives the $5 of real money at the end of the experiment will depend on the total amount they earn across the experiment. So make your choices carefully, keeping in mind what they'll mean both for you and for the other players.

At the end of the experiment, you'll be told your total earnings and whether you earned the $5 of real money. For each of the other two players, you'll also see how much was subtracted from your earnings because of the other player's choices. For example, Player A's final screen might look like this:

```
Earnings from your own choices:            + $___
Additional losses from Player B's choices:  − $___
Additional losses from Player C's choices:  − $___
                                            ──────
Your total earnings:                          $___

You didn't win the $5 in real money.
```

Do you have any questions?
